# TBP2 is a substitute for TBP in *Xenopus *oocyte transcription

**DOI:** 10.1186/1741-7007-7-45

**Published:** 2009-08-03

**Authors:** Waseem Akhtar, Gert Jan C Veenstra

**Affiliations:** 1 Department of Molecular Biology, Faculty of Science, Nijmegen Centre for Molecular Life Sciences, Radboud University Nijmegen, The Netherlands

## Abstract

**Background:**

TATA-box-binding protein 2 (TBP2/TRF3) is a vertebrate-specific paralog of TBP that shares with TBP a highly conserved carboxy-terminal domain and the ability to bind the TATA box. TBP2 is highly expressed in oocytes whereas TBP is more abundant in embryos.

**Results:**

We find that TBP2 is proteolytically degraded upon meiotic maturation; after germinal vesicle breakdown relatively low levels of TBP2 expression persist. Furthermore, TBP2 localizes to the transcriptionally active loops of lampbrush chromosomes and is recruited to a number of injected promoters in oocyte nuclei. Using an altered binding specificity mutant reporter system we show that TBP2 promotes RNA polymerase II transcription *in vivo*. Intriguingly, TBP, which in oocytes is undetectable at the protein level, can functionally replace TBP2 when ectopically expressed in oocytes, showing that switching of initiation factors can be driven by changes in their expression. Proteolytic degradation of TBP2 is not required for repression of transcription during meiotic maturation, suggesting a redundant role in this repression or a role in initiation factor switching between oocytes and embryos.

**Conclusion:**

The expression and transcriptional activity of TBP2 in oocytes show that TBP2 is the predominant initiation factor in oocytes, which is substituted by TBP on a subset of promoters in embryos as a result of proteolytic degradation of TBP2 during meiotic maturation.

## Background

A key regulatory step in eukaryotic transcription initiation is the assembly of basal transcription apparatus at the core promoter. This apparatus includes RNA polymerase II (pol-II) and a set of basal transcription factors. For a long time the basal transcriptional machinery was thought to be universal and mainly invariant across different promoters. However, a growing body of evidence points towards a more dynamic and regulatory role for this machinery (reviewed in [1-4]). TATA-box binding protein (TBP) was once thought to be the general transcription factor involved in all transcription in eukaryotic cells. In higher eukaryotes, however, a number of TBP paralogs are present and there is clear evidence for TBP-independent transcription in a variety of model organisms [5-12]. So far three TBP paralogs have been described in metazoans; the insect-specific TBP-related factor 1 (TRF1) [13], the metazoan-specific TBP-like factor (TLF, also known as TRF2, TLP or TBPL1) [1] and the vertebrate-specific factor TBP2 (also known as TRF3 or TBPL2) [8,12,14].

TBP2 is the most closely related TBP paralog, sharing 95% sequence identity in the core domain [8,12,14]. It can bind to TATA box, interacts with TFIIA and TFIIB and promotes basal transcription *in vitro*. In addition, knockdown studies in fish and frogs showed that TBP2 is indispensable for embryonic development [8,12] and is preferentially required for the transcription of embryonic vertebrate-specific genes and those involved in ventral specification during gastrulation in *Xenopus *[9]. TBP2 is also essential in differentiation pathways in zebrafish and mouse [10,11]. Although some TBP2 is present and required in early *Xenopus *embryos, it is most abundant in oocytes, suggesting that TBP2 has an important role in oocyte transcription [8]. Here we provide functional evidence for the role of TBP2 in pol-II transcription by examining its binding to oocyte chromosomes and pol-II promoters, and by employing *in vivo *transcriptional assays involving an altered binding specificity mutant reporter system. We show that TBP2 is localized to active promoters in oocytes and can promote pol-II transcription. TBP2 is degraded during meiotic maturation. Surprisingly, TBP, when exogenously expressed in oocytes, can substitute for TBP2, which is indicative of dynamic and rapidly adaptable nature of core transcription machinery. Together, these observations establish the involvement of TBP2 in transcription initiation of oocytes. Moreover the proteolytic degradation of TBP2 during meiotic maturation is relevant for the initiation factor switching that occurs during the course of early development.

## Methods

### Constructs

The *Xenopus *TBP (pSP64A-xTBP) [15] and TBP2 (pT7TSA-xTBP2) [8] constructs were utilized to generate altered binding specificity mutant versions of these proteins by introducing three point mutations in the carboxy-terminal domain of xTBP (I250F, V259T and L261V) and xTBP2 (I273F, V282T and L282V) using the Quick Change site-directed mutagenesis kit (Stratagene). Capped mRNAs were transcribed by an *in vitro *RNA synthesis kit (Ambion, Austin, TX). pCMV-CAT and pG4-hsp70-CAT have already been described [16] whereas pG4-BLCAT- ZFP36L2, pG4-BLCAT-TLF, pG4-BLCAT-polr2h and pG4-BLCAT-Cyr61 and pG4-BLCAT-intron (containing an intronic fragment of the WD repeat domain 42A (wdr42a) gene) were generated by cloning these promoters amplified from *X. tropicalis *genomic DNA, into pG4-BLCAT2 [17] at the *Bam*HI and *Xho*I sites (WA and GJCV, manuscript in preparation).

### Germinal vesicle spreads and immunofluorescence

Germinal vesicle (GV) spreads were prepared as described in [18] with minor modifications. Defolliculated stage VI oocytes were transferred one at a time to isolation medium (83 mM KCl, 17 mM NaCl, 6.5 mM Na_2_HPO_4_, 3.5 mM KH_2_PO_4_, 1 mM MgCl_2_, 1 mM dithiothreitol, pH 7.0). The GV was isolated, the nuclear membrane was removed and the contents of GV quickly transferred to 20 μl of spreading medium containing 1% paraformaldehyde and 0.15% Triton X-100 on a glass cover slip. The cover slips were kept at room temperature for 1 hr before being stored in phosphate-buffered saline (PBS). GV spreads were rinsed in PBS containing 0.2% Tween and blocked with 0.5% bovine serum albumin and 0.4% gelatin followed by 1 hr incubations with primary and secondary antibodies at room temperature. TBP2 antibody 1G6 has already been described [8] whereas 3E6 is a mouse monoclonal antibody raised against the amino-terminal domain of *Xenopus *TBP2. The secondary antibody was Alexa 488-labelled goat anti-mouse immunoglobulin G (IgG) in a 1:100 dilution also containing 4 μg/ml 4',6-diamidino-2-phenylindole (DAPI). Images were taken with Zeiss Axiophot2 Fluorescence microscope.

### Microinjections and primer extension

Defolliculated oocytes were injected with 1 ng of *in vitro*-transcribed TBP/2-abs RNA in the cytoplasm using a Drummond Nanoject microinjection apparatus (Drummond Scientific, Broomall, PA). After a delay of 4 hr, GVs of these oocytes were injected with different amounts of plasmid constructs as indicated in the text and figure legends. The next day the oocytes were collected and RNA was isolated using Trizol (Invitrogen) extraction and LiCl precipitation. The RNA equivalent to four oocytes was analyzed by primer extension as described in [17].

### Meiotic maturation and western blotting

Stage VI oocytes were incubated at 18°C in MBSH buffer containing 1 μg/ml progesterone (Sigma). Maturing oocytes were synchronized at germinal vesicle break down (GVBD) and then collected at different time points as indicated in the figure legends. Extracts from collected oocytes were prepared as previously described [15]. The antibodies used were anti-TBP 58C9, anti-TFIIB C-18, anti-TFIIF RAP 74 C-18, anti-Mos C237 (Santa Cruz Biotechnology), anti-phospho-p44/42 MAP Kinase (Cell Signalling Technology), anti-TBP SL27, SL30 and SL33 [19] and anti-H3 core and anti-H3Ser10P (Abcam).

### Chromatin immunoprecipitation

Stage VI oocytes were injected with promoter constructs (for amounts see the figure legends) in the nucleus. After overnight incubation at 18°C, oocytes were cross-linked and processed for chromatin immunoprecipitation (ChIP) as described previously [8]. For ChIP of HA-tagged TBP, 2 ng mRNA encoding HA-TBP2 was injected in the cytoplasm 4 hr prior to nuclear injections. The antibodies used were anti TBP-2 1G6 [8], anti-RNA polymerase II (Diagenode) and anti-HA (Covance).

### Electrophoretic mobility shift assay

An electrophoretic mobility shift assay (EMSA) was performed essentially as described in [20] and [21].

## Results

### Regulated proteolytic degradation of TBP2 during meiotic maturation

**Figure 1:**
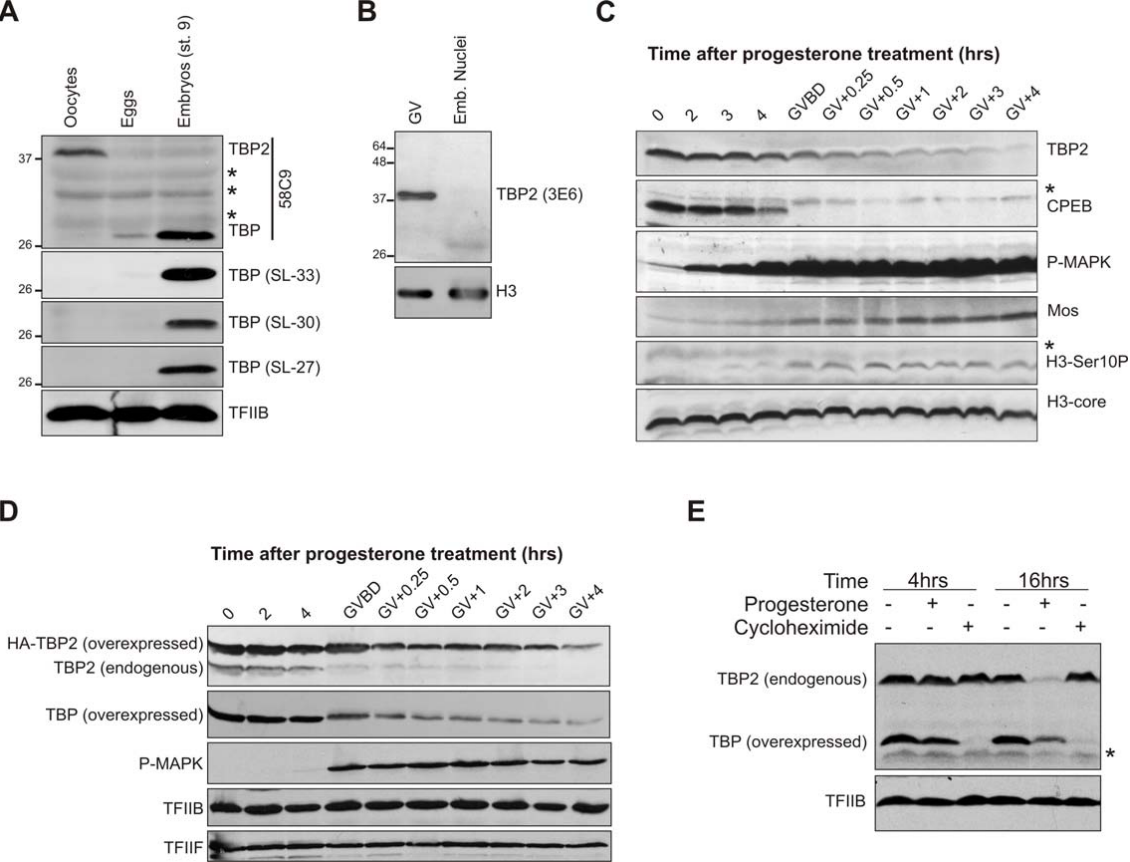
**TBP2 is actively down regulated upon meiotic maturation**. **(A) **Western blot analysis of TBP expression in oocytes, eggs and late blastula (stage 9) embryos with four different antibodies; 58C9, SL27, SL30 and SL33. Asterisk indicates non-specific bands. **(B) **Western blot analysis of TBP2 expression in oocyte germinal vesicles and embryonic nuclei with a TBP2-specific monoclonal antibody 3E6. **(C) **Stage VI *Xenopus *oocytes were incubated in MBSH buffer containing 1 μg/ml progesterone. Batches of 15 oocytes were collected at indicated time intervals and analyzed by western blotting. **(D) **Stage VI *Xenopus *oocytes injected with TBP and HA-TBP2 mRNA were incubated in MBSH buffer containing 1 μg/ml progesterone. Batches of 15 oocytes were collected at indicated time intervals and analyzed by western blotting. **(E) **Oocytes expressing exogenous TBP were treated with either progesterone (1 μg/ml) or cycloheximide (15 μg/ml). Batches of 15 oocytes were collected 4 hr and 16 hr after the treatment and analyzed by western blotting. Asterisk indicates non-specific bands.

TBP accumulates due to regulated translation of TBP mRNA during cleavage stages of development [15], whereas it is not detectable in oocytes (Figure 1A, see [8,15]). TBP2, by contrast, is very abundant in oocytes but is expressed at very low levels in embryos, suggesting that TBP2 is a replacement core promoter factor for TBP in oocytes [8,22]. To examine the possibility that the lack of detection of TBP in oocytes is due to a modification of TBP in these cells that masks its epitope, we probed oocyte (prophase of meiosis I-arrested), egg (metaphase of meiosis II-arrested) and late blastula (stage 9) embryo extracts with 58C9 antibody, which recognizes both TBP and TBP2, and three different TBP-specific antibodies, SL27, SL30 and SL33. None of the antibodies detect TBP in oocytes (Figure 1A), confirming that there is very little if any TBP present in *Xenopus *oocytes. Low levels of TBP are detected in egg extracts, whereas it is very abundant in embryos (Figure 1A), consistent with regulated translation of maternal TBP mRNA in cleavage-stage embryos [15].

TBP2 on the other hand is abundant in oocytes, whereas in eggs and early embryos very low levels of TBP2 are present. This was also observed when oocyte GV and embryonic nuclear extracts were probed with a TBP2-specific monoclonal antibody 3E6 (Figure 1B). The mechanism of the decrease in TBP2 expression, however, is unknown. To investigate this issue we followed the TBP2 levels during the course of oocyte maturation. This can be done *in vitro *by treating fully grown stage VI oocytes with progesterone. The maturation is marked by the appearance of a spot on the animal pole of the oocyte as a result of GVBD. TBP2 levels start to drop within 30 min after GVBD and by 4 hr the protein is hardly detectable (Figure 1C). This was further assessed by looking at some markers of meiotic maturation in oocytes, including degradation of cytoplasmic polyadenylation element binding protein and phosphorylation of MAPK and histone H3 serine10. TBP2 downregulation coincides with the accumulation of *Xenopus *Mos protein (Figure 1C). The Mos protein is a kinase that plays an important role in oocyte maturation. In response to progesterone treatment, the translation of Mos mRNA and the stability of the Mos protein increase, resulting in the gradual accumulation of the protein [23].

To examine the kinetics of overexpressed TBP and TBP2 in maturing oocytes, oocytes were injected with mRNA encoding TBP and HA-TBP2 (which can be distinguished from endogenous TBP2) and treated with progesterone. The injected synthetic TBP mRNA lacks its native 5' and 3'-UTR and is translated in oocytes, in contrast to the endogenous maternal TBP mRNA which is translationally masked [15]. The kinetics of overexpressed TBP and TBP2 are similar, although the levels of endogenous TBP2 tend to decrease more rapidly (Figure 1D), which may reflect differences in expression level at the start of the experiment.

To assess whether the decrease in TBP2 levels is due to changes in translation or degradation, oocytes were treated with the translation inhibitor cycloheximide. To check translation efficiency under these conditions oocytes were injected with TBP mRNA in the presence and absence of this inhibitor. After 16 hr of incubation with cycloheximide there was hardly any effect on TBP2 levels, showing that TBP2 protein is quite stable in oocytes, whereas in progesterone-treated cells TBP2 expression is lost (Figure 1E). Therefore, proteolytic degradation of TBP2 is markedly increased during meiotic maturation. TBP, when ectopically expressed in oocytes, is also degraded during maturation although residual levels after maturation are substantially higher, similar to the situation when TBP2 is overexpressed (Figures 1D and 1E). Intriguingly, even well after GVBD, TBP2 is not completely degraded, which is consistent with the observation that some TBP2 persists in early *Xenopus *embryos (Figure 1A, see [8]).

### TBP2 decorates lampbrush chromosomes

**Figure 2:**
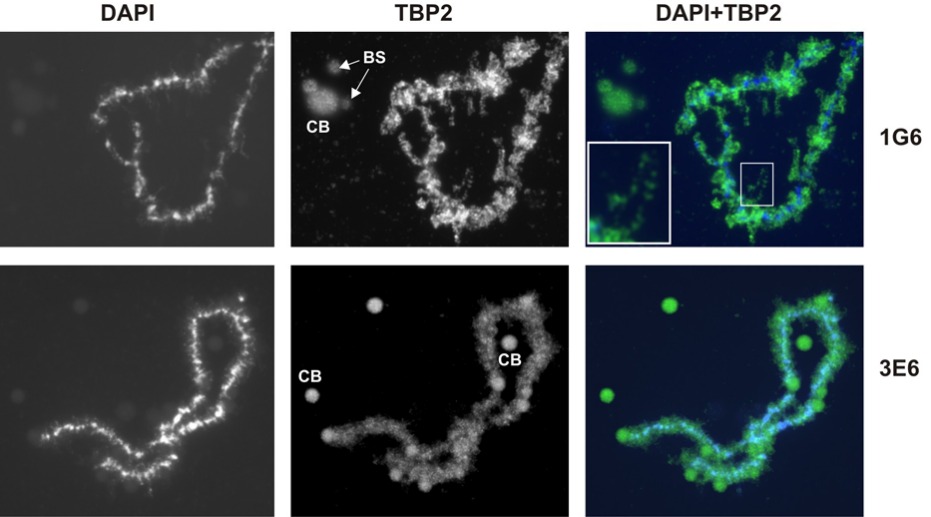
**TBP2 is recruited to transcriptionally active loops of lampbrush chromosomes**. Germinal vesical (GV) spreads were probed with TBP2 specific antibodies 1G6 (top panel) and 3E6 (bottom panel) as described in Methods. The inset shows a magnified chromosomal loop. Cajal bodies (CB) and B-snurposomes (BS) are indicated.

In the absence of TBP, TBP2 may be the major TATA box binding initiation factor in oocytes, and as such should localize to lampbrush chromosomes. These are partially condensed paired chromosomes present in the GVs of amphibian oocytes, arrested in the diplotene stage (prophase) of meiosis I. Transcriptional activity is accommodated by numerous laterally extended loops along the length of each of these chromosomes [24,25]. GV spreads were prepared from stage VI oocytes and the chromosome axis and the lateral loops were visualized using DAPI. Interestingly, many loops are decorated with TBP2 (Figure 2) as shown with two different TBP2 monoclonal antibodies. Nucleoli do not show any TBP2 staining; by contrast, Cajal bodies (CB) and B-snurposomes (BS), which are considered to be the depositories of transcription complexes and related proteins [18], stain positive. The two TBP2 antibodies used recognize different epitopes on TBP2 (data not shown) and show a similar staining of TBP2 on lampbrush chromosomes and CB (Figure 2). The recruitment of TBP2 to transcriptionally active chromosomal loops is in line with a major role of TBP2 in transcription initiation in oocytes.

### TBP2 is recruited to promoters in oocytes

**Figure 3:**
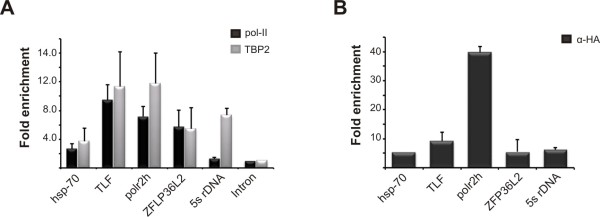
**TBP2 is associated with active promoters in oocytes**. **(A) **Chromatin immunoprecipitation (ChIP) assay on germinal vesical (GV)-injected promoters and endogenous 5S rRNA promoters using TBP2 and pol-II antibodies. Enrichment is the signal relative to background which in this case is the ChIP recovery from a non-promoter region (intron construct). **(B) **ChIP assay on GV-injected pol-II promoters and endogenous 5S rRNA promoter from oocytes injected with HA-TBP2 mRNA. Enrichment is the signal relative to background which in this case is the ChIP signal from oocytes not expressing HA-TBP2 but injected with the same promoter construct. Error bars in (A) and (B) represent the standard error of the mean from three independent experiments.

To characterize the binding of TBP2 at a higher resolution, promoter reporter constructs were injected into oocyte nuclei and analyzed by ChIP. As a control for the activity of these promoters we also performed ChIP with a pol-II antibody. The results show that TBP2 is associated with these promoters (Figure 3A). Although the recruitment is variable, in all cases it is well above the recruitment to the background control (construct containing an intronic fragment of the WD repeat domain 42A (*wdr42a*) gene). Furthermore, TBP2 recruitment correlates with the occupancy of pol-II. TBP2 was also enriched on the endogenous amplified 5S rRNA loci which recruited very little pol-II, as expected for RNA polymerase-III promoters.

To further confirm the association of TBP2 with promoters in oocytes, we expressed an HA-tagged version of TBP2 in oocytes and tested the recruitment of TBP2 on these promoters with an anti-HA antibody. Oocytes injected with promoter constructs but lacking HA-TBP2 were used as a negative control. A similar pattern of recruitment was obtained (Figure 3B). Together, these ChIP results show that TBP2 is recruited to pol-II and pol-III promoters in oocytes.

### Role of TBP2 in TATA box-dependent transcription in oocytes

**Figure 4:**
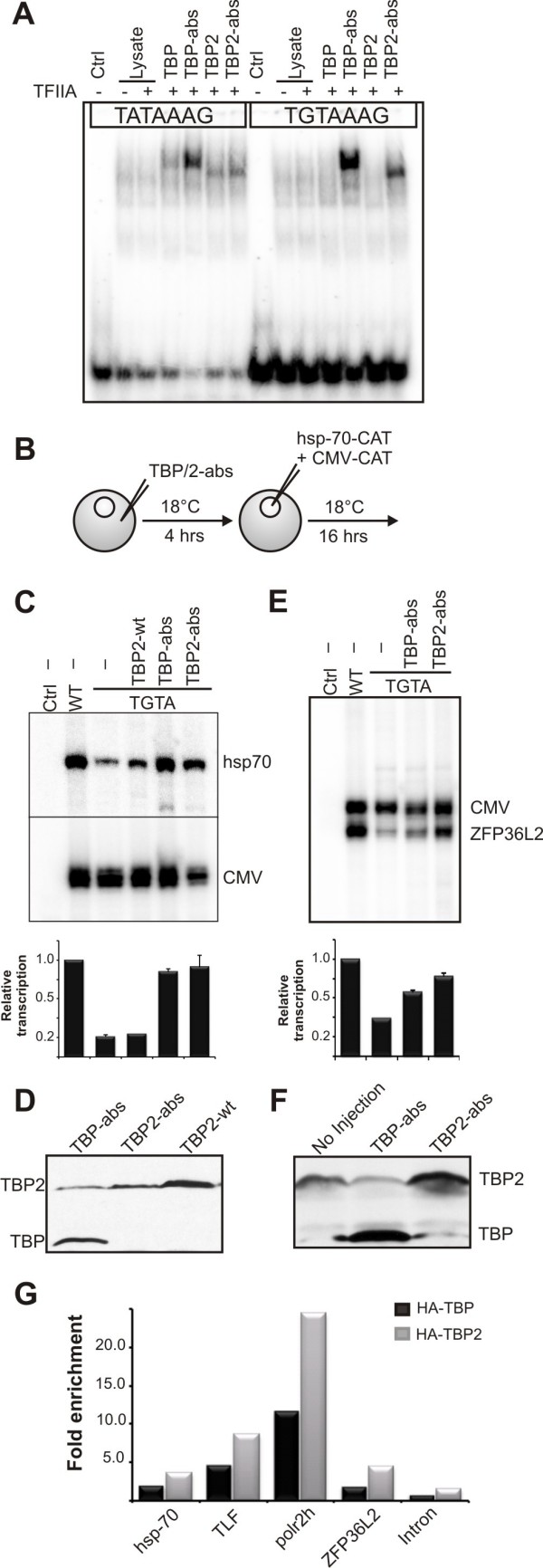
**Both TBP and TBP2 can promote transcription from TATA box containing promoters**. **(A) **EMSA showing the binding of *in vitro *translated wild-type and abs-mutant TBP and TBP2 to a wild-type and a mutant TATA box probe. **(B) **A scheme of the experiment shown in (C) is presented. TBP-abs or TBP2-abs mRNA was injected into stage VI oocytes. After 4 hr oocytes were injected with either wild type or TGTA mutant hsp-70 promoter. pCMV-CAT was injected as an internal control. **(C) **Primer extension was performed to determine the expression from the hsp70 (hsp-70) and CMV promoter (CMV). Ctrl: RNA from un-injected oocytes. The bar graph shows the quantification of transcription signal from hsp-70 promoter normalized with that from CMV. The error bars represent standard error of mean from three independent experiments. **(D) **Western blot analysis of TBP2-wt, TBP-abs and TBP2-abs expression in oocytes for the experiment in (C). **(E) **Expression from the ZFP36L2 promoter with regular or mutant TATA box in the presence of TBP and TBP2 abs-mutants was analyzed by primer extension. The bar graph shows the quantification of transcription signal from ZFP36L2 promoter normalized with that from CMV from two independent experiments. **(F) **Western blot analysis of TBP-abs and TBP2-abs expression in oocytes for the experiment in (E). **(G) **Chromatin immunoprecipitation (ChIP) assay on germinal vesical-injected promoters from oocytes injected with HA-TBP or HA-TBP2 mRNA. Enrichment is the signal relative to the ChIP signal from oocytes not expressing HA-tagged protein.

In order to further assess the role of TBP2 in oocyte transcription functionally, we applied an altered binding specificity mutant reporter system [26]. In this system, three point mutations in the carboxy terminal domain of TBP confer the ability to bind to a mutated TATA box (T**G**TAAAG) to which wild-type (wt) protein cannot bind. We introduced these altered binding specificity (abs) mutations in *Xenopus *TBP and TBP2. An EMSA was performed to assess the binding specificity of wt and abs-mutant TBP and TBP2. To this end, rabbit reticulocyte extracts containing *in vitro*-translated wt and abs-mutant versions of TBP or TBP2 were tested against regular and mutant TATA probes from the adenovirus major late promoter [20]. Like TBP, both wt and abs-mutant TBP2 proteins bind to a regular TATA box, whereas only the abs-mutant proteins bind to the altered TATA box (Figure 4A).

Next, we analyzed the behaviour of these proteins in oocyte transcription, for which we made use of the hsp70 promoter, which is known to be active in *Xenopus *oocytes and has a canonical TATA box upstream of its transcription start site [16]. The TATA box of this promoter was mutated to TGTA. Wild-type and the mutant promoters were injected in the oocytes with and without mRNA encoding TBP-abs or TBP2-abs (Figure 4B). Mutation of the TATA box reduces transcription from this promoter, although some transcription persists (Figure 4C). In the presence of TBP2-abs, transcription from the TGTA promoter is rescued. In contrast, wt TBP2 is unable to promote transcription from the TGTA promoter, confirming that the rescue is specifically carried out by the exogenously provided abs-mutant proteins. Interestingly, TBP-abs, when expressed in oocytes, also rescues the transcription to the same extent (Figure 4C) whereas the levels of exogenous abs proteins in both cases are comparable (Figure 4D).

We also tested the *X. tropicalis *ZFP36L2 promoter, which has a canonical TATA box positioned 27 bp upstream of the transcription start site. We generated the TGTA mutant of this promoter and used it to test the activity of TBP and TBP2 on this promoter. The TGTA mutation significantly reduces transcription from this promoter (Figure 4E). Both TBP-abs and TBP2-abs can rescue transcription, with TBP2-abs being slightly more efficient than TBP in this regard (Figure 4E).

Collectively, our findings show that TBP can substitute for TBP2 if provided artificially to the oocyte core transcription machinery. On the hsp70 and ZFP36L2 promoters, the relative expression levels of TBP and TBP2 appear to be a major determinant of initiation factor substitution. To analyze the behaviour of TBP and TBP2 on promoters lacking a TATA box, we examined the recruitment of exogenously provided HA-TBP and HA-TBP2 on two TATA-less promoters (TLF, polr2h) and two TATA box-containing promoters (hsp70, ZFP36L2). Oocytes injected with promoter constructs but lacking HA-TBP/2 were used as a negative control. Furthermore, a background control construct (intron) was injected. The results substantiate the notion that TBP, if ectopically expressed, can be recruited to these promoters in oocytes (Figure 4G).

### Loss of TBP2-promoter association during meiotic maturation

**Figure 5:**
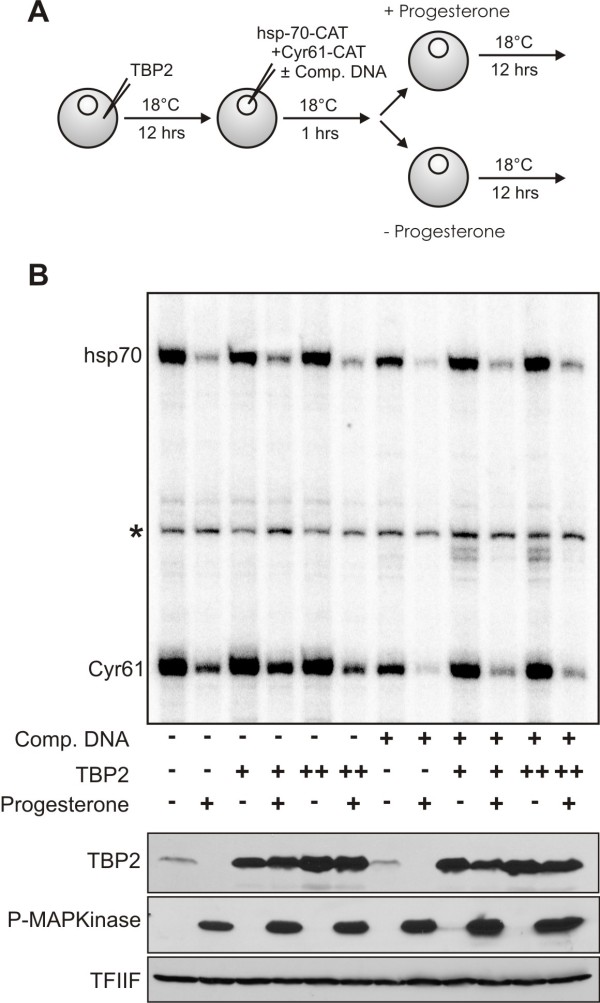
**Transcription repression occurs in the presence of abundant TBP2**. **(A) **A scheme of the experiment shown in (B) is presented. Either 2 ng or 4 ng of TBP2 mRNA was injected into stage VI oocytes. After a delay of 12 hr oocytes were injected in the nucleus with 1.0 ng of hsp70 and 0.5 ng of Cyr61 promoter constructs either with or without 18 ng of competitor DNA. One hour after the nuclear injections oocytes were divided into two groups. One group was treated with progesterone (final concentration 2 μg/ml). After a further incubation of 12 hr groups of 20 healthy oocytes were collected for RNA and protein analysis. **(B) **For transcription analysis, a primer extension was performed as described in Methods. The positions of accurately initiated transcripts from the hsp70 (hsp-70) and Cyr61 promoter (Cyr61) are indicated. The conditions used for each lane are described below the gel, and at the bottom expression of TBP2 has been shown for the corresponding lane. Phospho-MAPK was used as a marker for maturation while TFIIF served as a loading control.

In addition to degrading TBP2, maturing oocytes globally repress transcription. This prompted us to ask whether the downregulation of TBP2 may be important for proper shut down of transcription as oocytes mature into eggs. This is conceivable because TBP levels are extremely rate-limiting for transcription in egg extracts, and precocious transcription can be induced in embryos before gene activation at the mid-blastula transition by simultaneous inhibition of chromatin assembly with competitor DNA and premature accumulation of TBP [15,27]. To test this possibility, we overexpressed TBP2 in maturing oocytes. When TBP2 is overexpressed before maturation, the levels are also elevated after maturation (Figure 5B, bottom panel). Therefore, stage VI oocytes were injected with two different concentrations of TBP2 mRNA. The transcriptional shut-down was investigated on two distinct promoter constructs injected into oocytes either alone or in combination with high amounts of competitor DNA (Figure 5A). The results show that repression of transcription was established even in the presence of highly abundant TBP2 in maturing oocytes (Figure 5B, top panel). The overexpression of TBP2 or presence of competitor DNA, alone or in combination, had little if any effect on the repression of transcription.

**Figure 6:**
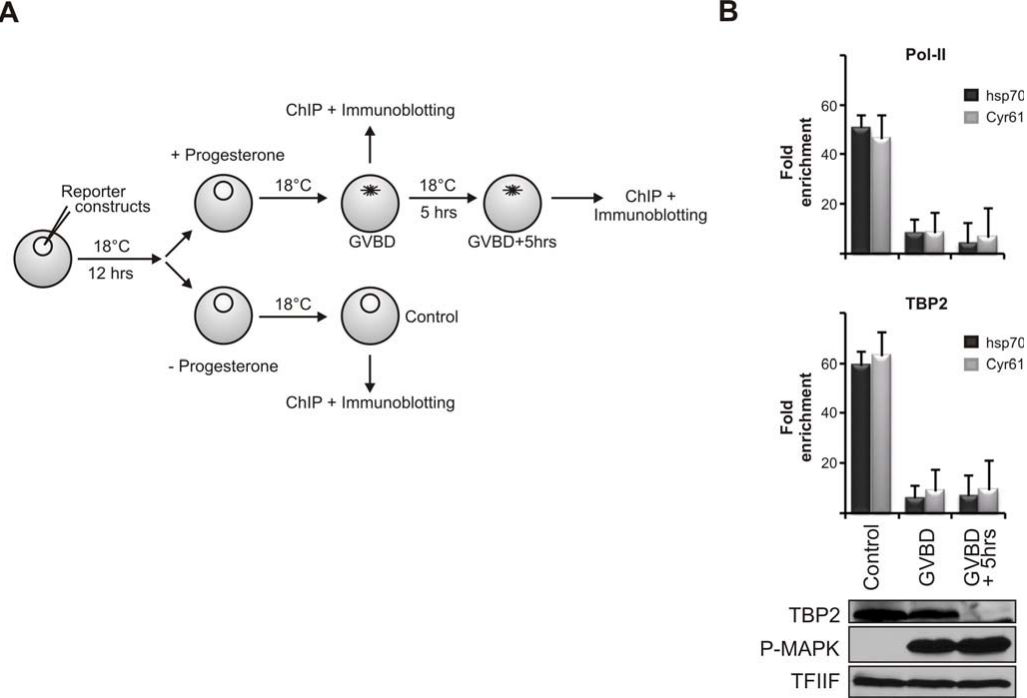
**TBP2 degradation takes place after the establishment of transcriptional shut down**. **(A) **A scheme of the experiment shown in (B) is presented. Oocyte nuclei were injected with 0.6 ng of hsp70 and 0.3 ng of Cyr61 promoter constructs. After a delay of 12 hr oocytes were divided into two groups and one group was treated with progesterone (final concentration 2 μg/ml). Oocytes were synchronized at germinal vesicle breakdown (GVBD). Some were processed immediately for chromatin immunoprecipitation (ChIP) and immunoblotting along with control oocytes (not treated with progesterone) whereas others were analyzed 5 hr after GVBD. **(B) **Recruitment of pol-II (upper graph) and TBP2 (lower graph) to hsp70 and Cyr61 promoters in control oocytes versus oocytes at GVBD and 5 hr after GVBD was assayed by ChIP. Below the graphs expression of TBP2 has been shown corresponding to each sample. TFIIF served as a loading control.

To further examine this situation we compared the timing of TBP2 degradation and the shutdown of transcription. Due to the variability between individual oocytes to undergo GVBD, batches of oocytes were synchronized depending on the appearance of the maturation mark at GVBD, and the promoter association of pol-II and TBP2 was determined by ChIP at GVBD and 5 hr after GVBD (Figure 6A). The results indicate that the repression of transcription is already established at GVBD, a point at which TBP2 degradation has not yet initiated. These observations (Figures 5 and 6) rule out a causative role for TBP2 degradation in the global shutdown of transcription during oocyte maturation, suggesting that the primary role of TBP2 degradation is to facilitate initiation factor switching during subsequent development. Not TBP2 degradation but rather a loss of TBP2 association with the promoter coincides with transcriptional repression during meiotic maturation.

## Discussion

### TBP2: the major TATA binding factor in oocyte transcription

In the present study we show that *Xenopus *oocytes lack any detectable TBP, whereas TBP2 is abundant in oocytes. In eggs and early embryos relatively low levels of TBP2 are present. TBP starts to accumulate after meiotic maturation and during cleavage stages of development (Figure 1A). A similar expression profile of TBP and TBP2 has been observed in mouse oocytes [22], which suggests a major role for TBP2 in oocyte transcription. Indeed, TBP2 is recruited to the transcriptionally active loops of lampbrush chromosomes (Figure 2). In addition, TBP2 also appears to be a part of CBs and BSs, which are considered to be involved in preassembly of transcription complexes [18]. The association of TBP2 with active promoters was further confirmed by ChIP on GV-injected promoters and on endogenous 5S rRNA (Figure 3). Surrounding follicle cells outnumber the oocytes; therefore it is not possible to perform ChIP on single copy loci in oocytes. Due to this limitation, injected promoter constructs were used to model oocyte transcription. This system has been used effectively to study the biochemistry of chromatin assembly and transcription during oogenesis and early embryogenesis [28]. In order to examine the activity of TBP2 in oocytes, we utilized an altered binding specificity (abs) mutant reporter system [26]. It cannot be ruled out that endogenous TBP2 behaves differently compared with its overexpressed abs mutant. However, the involvement of TBP2 in pol-II mediated transcription in oocytes may be inferred from the ability of abs-TBP2 to rescue the deleterious effects of TGTA promoter mutations, probably due to binding of abs-TBP2 to the TGTA sequence to which the endogenous TBP2 cannot bind (see Figure 4). These findings are congruous with the observation that only TBP2 is present in frog and mouse oocytes with no detectable TBP, which reinforces the notion that germ cell transcription is different from that of somatic cells [29].

### TBP2 degradation and its biological significance

We have found that during later stages of meiotic maturation TBP2 protein is actively degraded (Figure 1C). This could potentially achieve two biological goals. First, it might be essential for proper shutdown of global transcription. In early embryos, transcriptional repression can be perturbed by premature accumulation of TBP together with disruption of chromatin assembly [15,27]. Second, this degradation of TBP2 in combination with the translational upregulation of TBP in embryos might be a mechanism to achieve switching of TATA binding factors between the oocyte and embryonic and/or somatic transcription machineries. The results of our experiments do not support a role for TBP2 degradation in the shutdown of transcription as it occurs even in the presence of abundant TBP2 (Figure 5), although a redundant role in transcriptional repression cannot be excluded. Furthermore, transcriptional repression is already established before TBP2 is degraded (Figure 6). TBP2 degradation is likely to be a mechanism that brings about the switching of TATA binding core transcription factors, facilitating the transition from a germ cell transcription apparatus to a somatic transcription machinery. However, this switching is not yet complete in early embryos because of low levels of TBP2, as discussed below.

### The transcription machinery is highly flexible

The altered binding specificity mutant of TBP, when expressed in the oocytes, could also promote transcription from the hsp-70 and ZFP36L2 promoters (Figure 4). Earlier observations indicated that TBP and TBP2 can replace each other in initiating basal transcription *in vitro *as well as in complexes with TFIIA/ALF and DNA [8,30,31]. Intriguingly, many promoters that exclusively require TBP in embryos are also maternally expressed [9]. TBP protein, however, is not present in oocytes where these maternal transcripts were initially made, implying an initiation factor switching on these promoters between oocytes and early embryos. Furthermore, TBP2 overexpression in TBP knockdown embryos significantly, although not completely, rescued the phenotype of TBP ablation [8]. On a similar note, it has been proposed that in cells heterozygous for TBP (*tbp *+/-), elevated levels of TBP2 compensate abnormally low levels of TBP at some but not all promoters [32]. This shows that on many promoters the two TATA binding proteins can act redundantly.

A complicating matter is that both TBP and TBP2 are required for early development in fish and frog [5,6,8,12], and promoters that strictly require either TBP or TBP2 in embryos have been identified [9,10]. The molecular basis of TBP-TBP2 selectivity remains to be elucidated. The embryonic transcription machinery (abundant TBP, low levels of TBP2) is not yet fully switched to the common somatic machinery (abundant TBP, no TBP2), effectively leaving TBP and TBP2 to compete for interactions with embryonic activators and promoter elements. Paradoxically, despite the relatively low levels of TBP2 in embryos, many genes depend on this factor, whereas ablation of TBP causes relatively moderate effects on gene expression [9]. TBP2 may be competitive in embryonic gene regulation because of preferential interactions with embryonic activators or other maternal factors that – like TBP2 – still persist in early development; such preferential interactions would be analogous to the preference of the Caudal activator for a promoter nucleoprotein architecture with the downstream promoter element [33]. For example, TBP2 preferentially accommodates activation of vertebrate-specific embryonic and ventral-specific genes [9]. During later development, core factor switching may be completed when TBP2 levels further decline [31].

**Figure 7:**
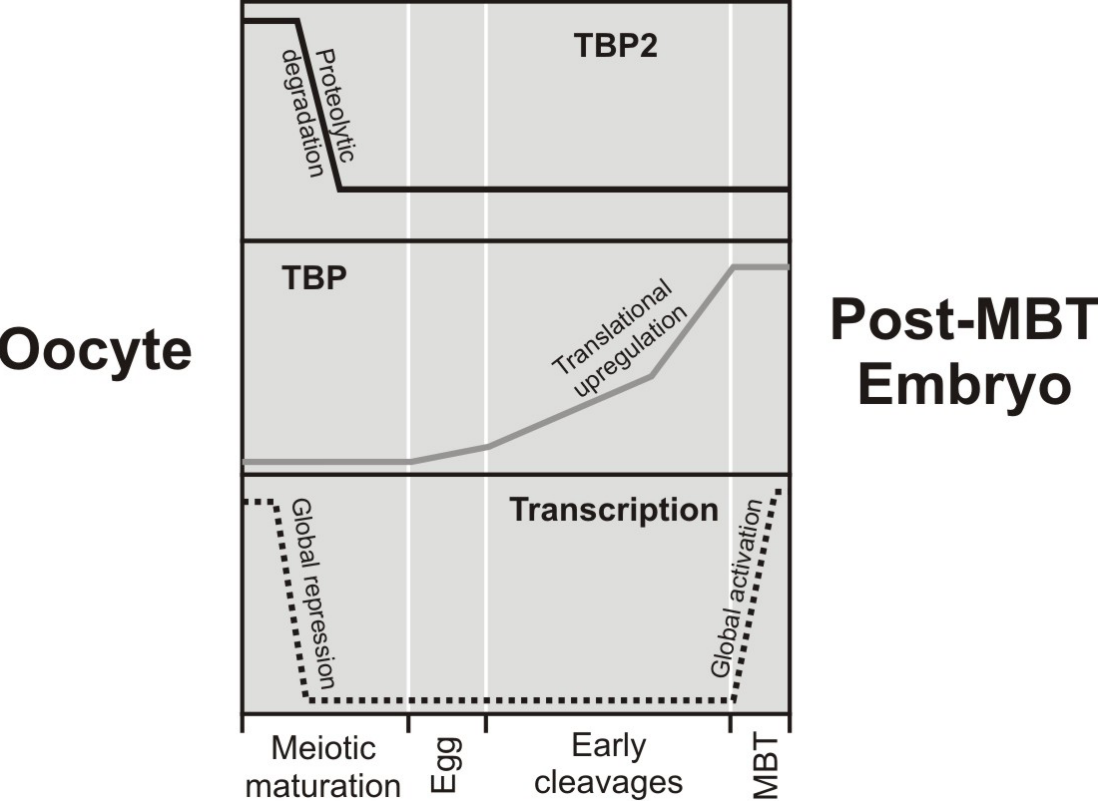
**A model of the regulation of TATA-box binding proteins during early stages of embryogenesis**. Oocytes express TBP2 which is involved in initiation of transcription. Upon meiotic maturation, global repression of transcription is established and TBP2 is actively degraded. During early cleavages after fertilization, maternal stores of TBP mRNA are translated [15] and by the mid-blastula transition (MBT) both TBP and residual TBP2 contribute to zygotic transcription [8,9].

On the limited number of promoters tested in this study, TBP and TBP2 could replace each other depending on their relative expression levels, providing an experimental model for initiation factor switching. Our data suggest that the core transcription machinery is highly flexible and that changes in relative expression levels of TATA binding factors between oocytes and embryos can drive core factor switching. Based on these observations we propose a model (Figure 7) according to which the core factor switching that occurs at many maternal-embryonic promoters is brought about by active degradation of TBP2 (Figures 1 and 2) followed by regulated translation of maternal stores of TBP mRNA before the onset of embryonic transcription at the mid-blastula transition (MBT) [15].

## Conclusion

Our observations not only establish an important role of TBP2 in transcription of a highly specialized cell type which lacks any detectable TBP, but also provide evidence that the basal transcription machinery is highly flexible and can rapidly exchange factors depending on their expression. This leads to initiation factor switching on a subset of promoters that are active both in oocytes and embryos, whereas other promoters show factor selectivity, which accounts for their non-redundant function in early embryos [8].

## Competing interests

The authors declare that they have no competing interests.

## Authors' contributions

WA designed and carried out the experiments, and drafted the manuscript. GJCV conceived of the study and contributed to its design and to writing the manuscript. Both authors read and approved the final manuscript.

## References

[B1] Dantonel JC, Wurtz JM, Poch O, Moras D, Tora L (1999). The TBP-like factor: an alternative transcription factor in metazoa?. Trends Biochem Sci.

[B2] Veenstra GJ, Wolffe AP (2001). Gene-selective developmental roles of general transcription factors. Trends Biochem Sci.

[B3] Hochheimer A, Tjian R (2003). Diversified transcription initiation complexes expand promoter selectivity and tissue-specific gene expression. Genes Dev.

[B4] Reina JH, Hernandez N (2007). On a roll for new TRF targets. Genes Dev.

[B5] Veenstra GJ, Weeks DL, Wolffe AP (2000). Distinct roles for TBP and TBP-like factor in early embryonic gene transcription in Xenopus. Science.

[B6] Muller F, Lakatos L, Dantonel J, Strahle U, Tora L (2001). TBP is not universally required for zygotic RNA polymerase II transcription in zebrafish. Curr Biol.

[B7] Martianov I, Viville S, Davidson I (2002). RNA polymerase II transcription in murine cells lacking the TATA binding protein. Science.

[B8] Jallow Z, Jacobi UG, Weeks DL, Dawid IB, Veenstra GJ (2004). Specialized and redundant roles of TBP and a vertebrate-specific TBP paralog in embryonic gene regulation in Xenopus. Proc Natl Acad Sci USA.

[B9] Jacobi UG, Akkers RC, Pierson ES, Weeks DL, Dagle JM, Veenstra GJ (2007). TBP paralogs accommodate metazoan- and vertebrate-specific developmental gene regulation. EMBO J.

[B10] Hart DO, Raha T, Lawson ND, Green MR (2007). Initiation of zebrafish haematopoiesis by the TATA-box-binding protein-related factor Trf3. Nature.

[B11] Deato MD, Tjian R (2007). Switching of the core transcription machinery during myogenesis. Genes Dev.

[B12] Bartfai R, Balduf C, Hilton T, Rathmann Y, Hadzhiev Y, Tora L, Orban L, Muller F (2004). TBP2, a vertebrate-specific member of the TBP family, is required in embryonic development of zebrafish. Curr Biol.

[B13] Crowley TE, Hoey T, Liu JK, Jan YN, Jan LY, Tjian R (1993). A new factor related to TATA-binding protein has highly restricted expression patterns in Drosophila. Nature.

[B14] Persengiev SP, Zhu X, Dixit BL, Maston GA, Kittler EL, Green MR (2003). TRF3, a TATA-box-binding protein-related factor, is vertebrate-specific and widely expressed. Proc Natl Acad Sci USA.

[B15] Veenstra GJ, Destree OH, Wolffe AP (1999). Translation of maternal TATA-binding protein mRNA potentiates basal but not activated transcription in Xenopus embryos at the midblastula transition. Mol Cell Biol.

[B16] Landsberger N, Wolffe AP (1995). Role of chromatin and *Xenopus laevis *heat shock transcription factor in regulation of transcription from the *X. laevis *hsp70 promoter *in vivo*. Mol Cell Biol.

[B17] Kass SU, Landsberger N, Wolffe AP (1997). DNA methylation directs a time-dependent repression of transcription initiation. Curr Biol.

[B18] Gall JG, Bellini M, Wu Z, Murphy C (1999). Assembly of the nuclear transcription and processing machinery: Cajal bodies (coiled bodies) and transcriptosomes. Mol Biol Cell.

[B19] Ruppert SM, McCulloch V, Meyer M, Bautista C, Falkowski M, Stunnenberg HG, Hernandez N (1996). Monoclonal antibodies directed against the amino-terminal domain of human TBP cross-react with TBP from other species. Hybridoma.

[B20] Maldonado E, Ha I, Cortes P, Weis L, Reinberg D (1990). Factors involved in specific transcription by mammalian RNA polymerase II: role of transcription factors IIA, IID, and IIB during formation of a transcription-competent complex. Mol Cell Biol.

[B21] Mitsiou DJ, Stunnenberg HG (2000). TAC, a TBP-sans-TAFs complex containing the unprocessed TFIIAalphabeta precursor and the TFIIAgamma subunit. Mol Cell.

[B22] Gazdag E, Rajkovic A, Torres-Padilla ME, Tora L (2007). Analysis of TATA-binding protein 2 (TBP2) and TBP expression suggests different roles for the two proteins in regulation of gene expression during oogenesis and early mouse development. Reproduction.

[B23] Haccard O, Jessus C (2006). Oocyte maturation, Mos and cyclins – a matter of synthesis: two functionally redundant ways to induce meiotic maturation. Cell Cycle.

[B24] Jamrich M, Warrior R, Steele R, Gall JG (1983). Transcription of repetitive sequences on Xenopus lampbrush chromosomes. Proc Natl Acad Sci USA.

[B25] Gall JG, Wu Z, Murphy C, Gao H (2004). Structure in the amphibian germinal vesicle. Exp Cell Res.

[B26] Strubin M, Struhl K (1992). Yeast and human TFIID with altered DNA-binding specificity for TATA elements. Cell.

[B27] Prioleau MN, Huet J, Sentenac A, Mechali M (1994). Competition between chromatin and transcription complex assembly regulates gene expression during early development. Cell.

[B28] Landsberger N, Wolffe AP (1995). Chromatin and transcriptional activity in early Xenopus development. Semin Cell Biol.

[B29] DeJong J (2006). Basic mechanisms for the control of germ cell gene expression. Gene.

[B30] Deato MD, Marr MT, Sottero T, Inouye C, Hu P, Tjian R (2008). MyoD targets TAF3/TRF3 to activate myogenin transcription. Mol Cell.

[B31] Xiao L, Kim M, DeJong J (2006). Developmental and cell type-specific regulation of core promoter transcription factors in germ cells of frogs and mice. Gene Expr Patterns.

[B32] Bush SD, Richard P, Manley JL (2008). Variations in intracellular levels of TATA binding protein can affect specific genes by different mechanisms. Mol Cell Biol.

[B33] Juven-Gershon T, Hsu JY, Kadonaga JT (2008). Caudal, a key developmental regulator, is a DPE-specific transcriptional factor. Genes Dev.

